# Hallazgos de tuberculosis extrapulmonar en imágenes de resonancia magnética en una paciente pediátrica

**DOI:** 10.7705/biomedica.5857

**Published:** 2021-10-15

**Authors:** Hamilton Delgado-Argote, Luz Miriam Leiva, Christian Rojas

**Affiliations:** 1 Facultad de Ciencias de la Salud, Universidad Icesi, Cali, Colombia Universidad Icesi Facultad de Ciencias de la Salud Universidad Icesi Cali Colombia; 2 Departamento de Medicina Física y Rehabilitación, Hospital Universitario del Valle, Universidad del Valle, Cali, Colombia Universidad del Valle Universidad del Valle Cali Colombia; 3 Neurología Infantil, Departamento de Pediatría, Hospital Universitario del Valle, Universidad del Valle, Cali, Colombia Universidad del Valle Universidad del Valle Cali Colombia

**Keywords:** tuberculosis, tuberculosis meníngea, tuberculosis osteoarticular, niño, Tuberculosis, tuberculosis, meningeal, tuberculosis, osteoarticular, child

## Abstract

La tuberculosis pulmonar es la más común en niños y su forma extrapulmonar corresponde aproximadamente a 30 a 40 % de los casos.

Se presenta el caso de una niña indígena con tuberculosis diseminada: pulmonar, cerebral, medular y musculoesquelética, con importantes secuelas en el neurodesarrollo. Este caso ilustra el espectro de la tuberculosis extrapulmonar pediátrica en países endémicos en desarrollo. Además, evidencia la gravedad de las complicaciones neurológicas causantes de grave discapacidad y resalta el valor de las imágenes radiológicas para orientar la sospecha diagnóstica de compromiso extrapulmonar.

## Abstract (en)

Pulmonary tuberculosis is the most common in children and its extrapulmonary manifestations are present in 30% to 40% of cases.

We present the case of an indigenous girl with disseminated tuberculosis: pulmonary, brain, medullary, and musculoskeletal with substantial neurodevelopmental sequelae. This case exemplifies the spectrum of pediatric extrapulmonary tuberculosis in endemic developing countries. Furthermore, it shows the severity of highly disabling neurological complications and stresses the importance of radiological imaging in guiding diagnostic suspicion of extrapulmonary involvement.

La tuberculosis pulmonar es el tipo más común de la enfermedad en los niños; la forma extrapulmonar se presenta en 30 a 40 % de los casos ^1^^,^^2^. Colombia tiene una incidencia intermedia de cerca de 25 casos por cada 100.000 habitantes ^3^. La población infantil es más vulnerable frente a la tuberculosis diseminada y extrapulmonar; y son factores de riesgo ser menor de 5 años, presentar inmunodeficiencias, no estar vacunados con la BCG y la desnutrición ^4^^,^^5^, la cual constituye el principal factor de riesgo en los países endémicos en desarrollo ^6^.

En Colombia, el compromiso extrapulmonar, que es el que más morbimortalidad genera, se ha documentado en el 34 % de los casos de tuberculosis en niños ^3^. En países como Uruguay, la forma de presentación más frecuente en menores de 15 años es la pleural, la cual ocurre mayoritariamente en niños previamente sanos ^4^. El sistema linfático es el más afectado ^3^, en tanto que la infección diseminada y la afectación del sistema nervioso central son las manifestaciones más frecuentes en pacientes inmunocomprometidos ^7^.

El compromiso del sistema nervioso central es la complicación más importante, causante de grave discapacidad y de tasas de mortalidad entre el 15 y el 32%, en tanto que el 80% de los sobrevivientes presenta secuelas neurológicas ^6^. En México, se ha documentado que la incidencia de este tipo de compromiso es del 1% del total de casos y de 6 a 10% de los extrapulmonares en pacientes inmunocompetentes, siendo la manifestación más frecuente la meningitis, seguida por el tuberculoma y el absceso tuberculoso ^8^.

En la leptomeningitis tuberculosa, el estudio mediante imágenes permite detectar el realce paquimeníngeo basal, la hidrocefalia no obstructiva y el infarto parenquimatoso, hallazgos muy específicos para la meningitis por tuberculosis ^7^^,^^9^, cuyas características radiológicas varían según el momento de la evolución ^7^. A menudo, la radiculomielitis es una extensión de la tuberculosis meníngea que afecta la médula espinal y las raíces nerviosas, lo que en el estudio radiológico se evidencia por el realce meníngeo irregular perimedular y radicular ^7^^,^^10^.

La manifestación osteomuscular más frecuente es la espondilitis tuberculosa, seguida de la artritis séptica, la cual suele ser monoarticular y comprometer la cadera o la rodilla, lo que se manifiesta radiológicamente como sinovitis ^7^. Otras afecciones menos frecuentes son la tenosinovitis y la miositis por tuberculosis.

En este artículo se presentan las imágenes de resonancia magnética (RM) de una paciente con tuberculosis extrapulmonar extensa.

## Caso clínico

Se trata de una niña indígena de 9 años de edad, residente en un resguardo en el Valle del Cauca, con antecedentes de desnutrición e historia de desarrollo psicomotor acorde para la edad; al no presentar el carné correspondiente, se asumió que no había sido vacunada con BCG.

Consultó por un cuadro de 14 días de fiebre, dolor abdominal difuso y emesis. En el examen físico, presentaba fiebre (40°c), taquicardia (120 latidos por minuto), desnutrición (peso para la talla de menos de dos desviaciones estándar), periodos fluctuantes de somnolencia e irritabilidad, pero sin signos meníngeos. No se evidenciaron cambios inflamatorios o infecciosos en oídos ni orofaringe y, tampoco, lesiones en la piel.

En los exámenes de laboratorio, se reportó leucocitosis, elevación de la proteína C reactiva, hipocaliemia e hiponatremia; la IgG contra citomegalovirus fue positiva y, la IgM, negativa, la IgG y la IgM para toxoplasma, leptospira y dengue, así como los antígenos febriles, la gota gruesa y los hemocultivos, fueron negativos.

La ecografía de abdomen fue normal y no se identificaron imágenes nodulares, miliares, ni masas o áreas de consolidación en la radiografía de tórax.

Durante la evolución, presentó deterioro neurológico con episodios convulsivos tónico-clónicos e inestabilidad hemodinámica progresiva, por lo cual se le dio ingreso a la unidad de cuidados intensivos por impresión diagnóstica de neuroinfección. Se le hizo una punción lumbar y el líquido cefalorraquídeo (LCR) se encontró ligeramente turbio, con presión de apertura elevada, predominio linfocítico, hipoglucorraquia, y proteinorraquia, en tanto que el panel meníngeo (FilmArray^™^) fue negativo, así como la coloración Gram y la prueba de BAAR.

Se confirmó el diagnóstico de tuberculosis con base en las manifestaciones clínicas, el nexo epidemiológico (procedente de zona endémica), los factores de riesgo (desnutrición, sin inmunización) y la prueba molecular Xpert MTB/RIF^™^ positiva en el LCR y la secreción traqueal, con cultivo de LCR positivo para tuberculosis sensible a isoniazida y rifampicina.

Dados los episodios convulsivos, la postura antálgica en flexión de la cadera y la rodilla derecha con limitación para la rotación pasiva de la cadera, la presencia de alodinia y la hiperalgesia a partir del dermatoma a nivel de C4, se hizo la resonancia magnética (RM) de cerebro, columna total y pelvis.

**Figura 1:**
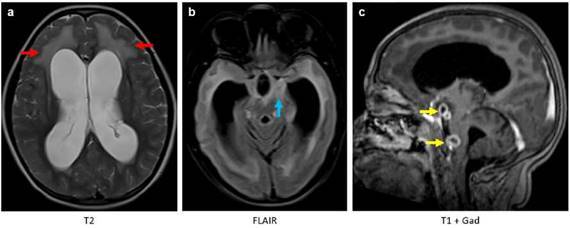
Resonancia magnética cerebral. Imágenes axiales potenciadas en T2 **(a)**, FLAIR **(b)** y coronales T1 después del uso de gadolinio **(c)**. Hidrocefalia no obstructiva supra e infratentorial con hiperintensidades de la sustancia blanca periventricular (flecha roja) correspondiente a edema transependimario por actividad hidrocefálica. Aumento de la intensidad de señal en las meninges de la base del cráneo (flecha azul) como causa principal de la hidrocefalia por obstrucción del flujo cefalorraquídeo y disminución en su absorción. Se detectaron dos lesiones extraaxiales con realce en anillo, correspondientes a focos de tuberculomas (flechas amarillas).

**Figura 2:**
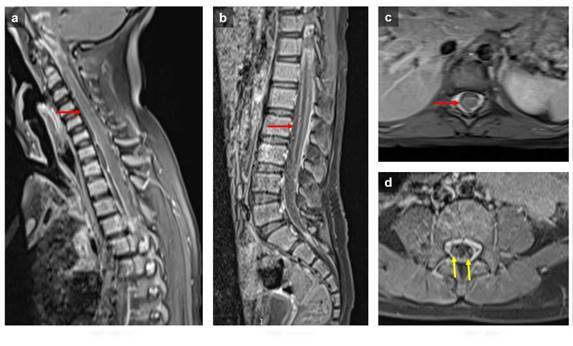
Resonancia magnética de columna. Imágenes sagitales **(a y b)** y axiales a nivel torácico **(c)** y lumbar **(d)** potenciadas en T1 después del uso de gadolinio. Realce meníngeo en los segmentos cervicales, torácico y lumbar (flecha roja) con realce de las raíces (flecha amarilla)

**Figura 3:**
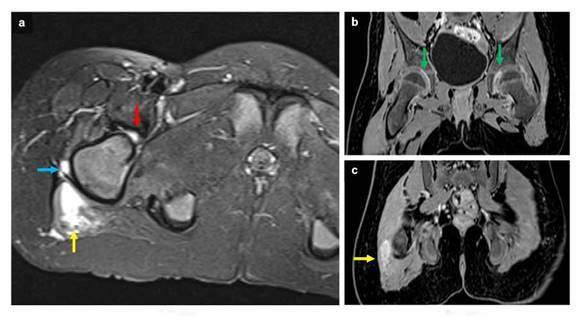
Resonancia magnética de pelvis. Imagen axial potenciada en T2 con saturación grasa **(a)** y coronales potenciada en T1 después del uso de contraste **(b y c)**. Edema del margen lateral del musculo glúteo mayor derecho (flecha amarilla) y de vasto lateral del cuádriceps ipsilateral (flecha azul). Nótese la presencia de derrame articular coxofemoral (flecha roja) con realce después del uso de contraste indicativo de sinovitis bilateral (flecha verde).

Las imágenes obtenidas evidenciaron tuberculosis cerebral, con hallazgo de meningitis, tuberculomas e hidrocefalia (figura 1), tuberculosis medular con presencia de colecciones leptomeníngeas y radiculitis (figura 2), y tuberculosis osteomuscular con sinovitis, bursitis y miositis (figura 3).

La niña recibió tratamiento con isoniacida, rifampicina, pirazinamida y etambutol (fases 1 y 2) durante seis meses, y con prednisolona, levetiracetam (como anticonvulsivante), morfina y amitriptilina para el manejo del dolor somático y el neuropático, y baclofeno y clonazepam para tratar la espasticidad.

Se descartó una inmunodeficiencia primaria o adquirida (HIV no reactivo, niveles de inmunoglobulinas y complemento normales). Sin embargo, presentó una evolución tórpida, con secuelas neurológicas muy incapacitantes por la lesión cerebral extensa (deterioro cognitivo, espasticidad, movimientos anormales y epilepsia), lo que significó dependencia en todas las actividades de autocuidado, pues hubo necesidad de ventriculostomía externa, gastrostomía y traqueostomía. Además, la paciente tuvo secuelas osteomusculares, entre ellas, contracturas articulares, retracciones en cadera y rodilla, y dolor neuropático atribuido a radiculitis.

En una junta interdisciplinaria, se decidió que la paciente debía recibir cuidado paliativo y se le dio egreso con un programa de cuidado en casa.

El diagnóstico de tuberculosis extrapulmonar constituye un reto y el estudio con imágenes permite orientar el diagnóstico ^1^^,^^7^.
